# The Effect of Freezing and Thawing on Complex Permittivity of Bovine Tissues

**DOI:** 10.3390/s22249806

**Published:** 2022-12-14

**Authors:** Anđela Matković, Antonio Šarolić

**Affiliations:** FESB, University of Split, HR-21000 Split, Croatia

**Keywords:** complex permittivity measurement, open-ended coaxial probe, freezing and thawing of biological tissues, ex vivo bovine tissues, brain white and grey matter, muscle, liver, microwave frequency range

## Abstract

The aim of this study was to investigate how the freezing and thawing of biological tissues affect their complex permittivity in the microwave frequency range from 0.5 MHz to 18 GHz. We measured the complex permittivity of ex vivo bovine tissues, including brain white and grey matter, liver, and muscle, using an open-ended coaxial probe. Bovine tissues were chosen for their availability and similarity to human tissue permittivity. The samples were measured at 25 °C, before they were frozen either in a commercial freezer below −18 °C or in liquid nitrogen, nominally at −196 °C. The measured permittivity before freezing was compared to the permittivity measured after freezing and thawing the tissues back to 25 °C. Statistical analysis of the results showed a statistically significant change in permittivity after freezing and thawing by both methods for all the measured tissues, at least in some parts of the measured frequency range. The largest difference was observed for the white matter, while the liver had the smallest percent change.

## 1. Introduction

The number of applications of electromagnetic fields expands, both in the biomedical and wireless communications domains. Accordingly, there is a growing demand for the exact knowledge of the complex permittivity of human tissues in the widest possible frequency range. This requires new and extended measurement studies on human tissues. However, in vivo measurements of human internal organs are either unrealizable or are hindered by many difficulties; thus, ex vivo measurements are often performed, providing an obvious alternative. Furthermore, due to the limited availability of human tissue samples, the tissues of large mammals are often measured instead, and the obtained permittivity values are considered appropriate to model human tissues as well [1,2].

The motivation for this work came from our previous studies [1,3], which reported the results of the dielectric measurement campaign on excised human brain tissues obtained from hospital surgeries and autopsies. The timing of the samples’ arrival was subordinate to the hospital schedule, while the measurement throughput was determined by the measurement logistics. Consequently, occasionally a problem would arise with the harmonization of the two throughputs, creating the need to store the tissue samples for a certain amount of time. Naturally, this requires resolving the issue of tissue degradation with time. During the said studies, tissue degradation was observed, both visibly on the tissue and on the complex permittivity measurement results, as dehydration and decomposition took place. Therefore, the studies reveal the need for tissue preservation, preferably over an extended period of time.

Freezing is a common method of preserving tissue from decomposition. It has even been used in the studies of tissue electric properties, such as in [4], where the tissue samples were initially stored in a freezer at −80 °C and subsequently thawed to room temperature before measurement. In the study [4], the samples were resected brain tumor tissues obtained from human patients. However, given that there were no published studies analyzing the effect of freezing and thawing on the conductivity of brain tissues or brain tumors, the authors could not confidently state that the tissue freezing and thawing process did not affect the measured conductivity.

Future extensive biological tissue measurement campaigns (aiming, e.g., to extend the frequency range of healthy tissues or to extend the knowledge of pathological tissues) would certainly benefit from a preservation protocol. Therefore, it is important to investigate how tissue freezing and thawing affects its complex permittivity. Due to differences in tissue composition, studies on the effects of freezing and thawing should be performed separately for different types of tissues.

Previously we reported the preliminary results of complex permittivity measurements of bovine and porcine brain and bovine liver before freezing in a commercial freezer and after thawing [5]. Here, we present a complete study focusing on bovine tissues only: the brain, liver, and muscle. Bovine tissues were chosen due to the availability of large healthy tissue samples. We expected that the changes in the bovine tissues’ permittivity that would occur as a result of the freezing and thawing protocol would similarly influence the human tissues. Our recently published study [1] shows that the complex permittivity of bovine and human white matter and grey matter is very similar. As another example, a recent paper investigated the thermal and frequency dependence of dielectric properties of ex vivo liver tissue [6] from the aspect of microwave ablation medical procedures, measuring the bovine liver as well.

The issue of permittivity preservation after the freeze–thaw cycle was approached by a recent study [7] for three tissues: bovine liver, bovine fat, and chicken muscle. The permittivity of the three tissues was measured at room temperature, before and after freezing and thawing, in the frequency range of 0.5–8.5 GHz. The statistical analysis was performed at only three discrete frequencies. The study found significant differences in both *ε*′ and *ε*″ for the bovine muscle at three analyzed frequencies (2.5, 4.5, and 6.5 GHz). The bovine liver had significant differences in the imaginary part for all three frequencies but only at 2.5 GHz for *ε*′. For the bovine fat tissue, *ε*′ slightly decreased, and *ε*″ changed insignificantly, but both changes were not statistically significant. The authors concluded that the freeze–thaw cycle results in an increase in the complex permittivity for high-water content tissues, while low-water content tissues did not have a significant change in complex permittivity.

In comparison with the aforementioned study, we introduced liquid nitrogen as an additional fast-freezing method and expanded the upper-frequency limit to 18 GHz. Furthermore, we reported both the results and the statistics continuously in the whole frequency range instead of just in several discrete frequency points. Extending the frequency range is important due to the expansion of both medical and wireless communication technologies to ever higher frequencies. Unfortunately, in this study, we were limited to 18 GHz by the available instrumentation. The chosen microwave frequency range is highly utilized in various medical applications, from diagnostics (e.g., cancer detection and microwave imaging) to treatment (e.g., microwave ablation and medical hyperthermia), as well as in all generations of wireless technologies.

Another distinction of this study is the introduction of brain tissues into the experiment, as the brain tissue was the primary interest of our human tissue measurement campaign [1]. The muscle and liver are rather homogenous tissues that could serve well for additional investigation and control of the freezing and thawing method. On the other hand, to the best of our knowledge, the effect of freezing on the permittivity of brain tissues has never been previously studied, which makes this the first study to approach this topic. Additionally, we did not find any published references that examined the permittivity changes of biological tissues after freezing and thawing using liquid nitrogen.

## 2. Materials and Methods

### 2.1. Sample Handling and Preparation

Dielectric properties of a material are described by its complex dielectric permittivity:

*ε* = *ε*′ − i*ε*″. (1)

The real part *ε*′ expresses the ability of the material to store the electric energy, while the imaginary part *ε*″ denotes the losses in the material, comprising of the material conductivity and the dielectric losses. We measured *ε*′ and *ε*″ of ex vivo bovine tissues at the room temperature of 25 °C, first on the fresh ex vivo samples, then on the same samples after they were frozen and thawed back to the room temperature of 25 °C.

We acquired two entire bovine brains, two entire lobes of bovine livers from two different specimens, and two bovine muscles from two different specimens from a local butcher. After the initial measurements on fresh samples, they were either frozen below −18 °C with the use of a commercial freezer or frozen by immersing in liquid nitrogen, nominally at −196 °C. The bovine brain, muscle, and liver that were frozen and stored in the freezer are labeled as B1, M1, and L1, respectively, while the ones that were frozen and stored in the liquid nitrogen tanks are labeled as B2, M2, and L2.

Before bringing the samples to the lab, all the samples were kept fresh in the butcher’s store for ca. 1 day after slaughter by refrigeration at 1 °C. The samples were immediately dissected upon bringing them to the lab. Both brains were first halved into the left and right hemispheres and were then dissected into coronal slices ca. 1.5 mm thick. Bovine liver and muscle were cut into 3 × 3 × 2 cm^3^ cuboids, which were visually entirely homogeneous. The size of the samples was chosen to obtain a maximum number of samples, each of them sufficiently large to satisfy the minimum sample size recommendation by the coaxial probe manufacturer (covered in Section 2.2) and to provide the space for multiple measurement points on each sample. Upon dissection, the cut samples were immediately stored in separate labeled airtight plastic containers and put in the refrigerator at 3 °C while they were waiting to be measured. Samples were sequentially taken out of the refrigerator and, while still in their sealed containers, heated to 25 °C with the help of the water bath. Each sample was measured at multiple points at 25 °C and then immediately put in a sealed container, either in the freezer, below −18 °C, or in the liquid nitrogen container, at −196 °C. The samples were left to freeze for 3 days and were then thawed back in the water bath set to the room temperature of 25 °C, and their permittivity was then measured again. Finally, the results were processed to compare the permittivity at the same temperature of 25 °C before freezing and after thawing.

### 2.2. Dielectric Measurement Setup

Measurements were carried out using FieldFox N9927A vector network analyzer (VNA) by Keysight Technologies (Santa Rosa, CA, USA) [8] connected to the Slim Form open-ended coaxial probe from Keysight’s N1501A Dielectric Probe Kit (Keysight Technologies Inc., Santa Rosa, CA, USA) [9] by a phase-stable coaxial cable Sucoflex 404 (HUBER+SUHNER AG, Herisau, Switzerland). The VNA was connected to a computer where the measurements were performed using the manufacturer-provided software Keysight Materials Measurement Suite N1500A (Keysight Technologies Inc., Santa Rosa, CA, USA) [10]. The room and the tissue sample temperature during measurements were kept at 25 °C, and the temperature of the material under test was controlled using a precise thermometer DTM3000 (LKM Electronics GmbH, Geratal, Germany).

The measurement frequency range was limited at the upper limit by the VNA to 18 GHz, while the lower limit was 500 MHz, set by the Slim Form Probe lower frequency limit. Each measurement included 3501 linearly spaced frequency points from 500 MHz to 18 GHz. Several such measurements were performed on each tissue sample, moving the probe to different measurement points on the sample. The Slim Form Probe’s outer diameter was just 2.2 mm, which allowed measuring of several points on each sample and distinguishing the tissue type on an inhomogeneous brain slice containing both the white and the grey matter. For these reasons, we did not use any wider probes.

The standard calibration procedure was performed with open, short, and deionized water measurements prior to each set of measurements. The probe was cleaned with 70% ethyl alcohol between the measurements to prevent cross-contamination and ensure the best results. After cleaning the probe with alcohol, we waited until the alcohol evaporated so as to not influence the results. The setup was recalibrated at regular intervals with deionized water.

**Figure 1:**
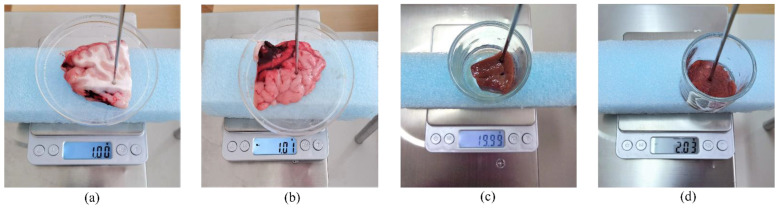
Measurement setup, including the dish with the sample supported by polystyrene on top of a weight scale for bovine (**a**) brain white matter, (**b**) brain grey matter, (**c**) liver, and (**d**) muscle.

The coaxial probe was fixed on the probe stand, and its open end was put in contact with a biological tissue sample. The sample was contained in a dish supported by thick layers of polystyrene foam on top of a precise weight scale. The scale was situated on top of a metal laboratory jack for *z*-axis adjustment so that the samples were lifted to the probe without disrupting the stability of the measurement setup. We did not notice any effect of the support structure on the measurement instability or inaccuracy. The measurement setup with each sample is shown in Figure 1.

Keysight recommends that the probe must be immersed in the material under test and surrounded by at least 5 mm of the material on all sides. Immersion is only attainable in liquid materials, as recommended by the manufacturer, while the usual practice when measuring biological tissues is to firmly press the probe against the tissue surface. Nevertheless, the liver samples were thick and homogeneous enough to allow the probe insertion into a sample by creating a tight insertion hole and achieving a configuration of the probe inserted into a homogeneous sample of the single tissue type. A similar approach was applied in [6], where the probe was inserted approximately 5 mm into the liver tissue. The inhomogeneous structure of the brain slices did not allow us to insert the probe as it would not be surrounded by only one tissue type (either white matter or grey matter) in sufficient volume; thus, the probe was just firmly pressed against the brain slice surface, either on the white matter or grey matter. The muscle samples, although homogenous, were too firm to successfully puncture with the coaxial probe without the severe compression of the sample, which would alter the results; therefore, the probe was only pressed against the muscle samples.

The precise weight scale in the setup was included to control the pressure that the probe exerted on the samples. This pressure was held constant as much as possible for each tissue type by fine adjustments of the laboratory jack height during the measurements, keeping the constant weight on the scale. The weight was kept at ca. 20 g after puncturing the liver, which translates to the constant pressure of ca. 50 kPa. The muscle samples were pressed with ca. 5 kPa, while both the white and grey matter were measured with the constant pressure of ca. 2.5 kPa, as the brain tissue was the most pliable of the three. These values were determined for each tissue type by observations to ensure firm contact between the probe and the tissue while avoiding significantly deforming the tissue, which depends on each tissue’s plasticity and elasticity.

When performing measurements on the brain tissue, the measurements were performed on the homogeneous regions of either white or grey matter when such regions were clearly visible on the brain slice. It is worth noting that not all of the slices displayed such properties; thus, some of the slices were discarded. Additionally, the regions with visually undifferentiated inhomogeneities were avoided. An example of an acceptable region of white and grey matter for performing the measurements is shown in Figure 1a,b, respectively.

## 3. Results

The results were processed as the average of all the measurement points on all samples of the same tissue type, thus increasing the statistical strength of the results.

**Figure 2:**
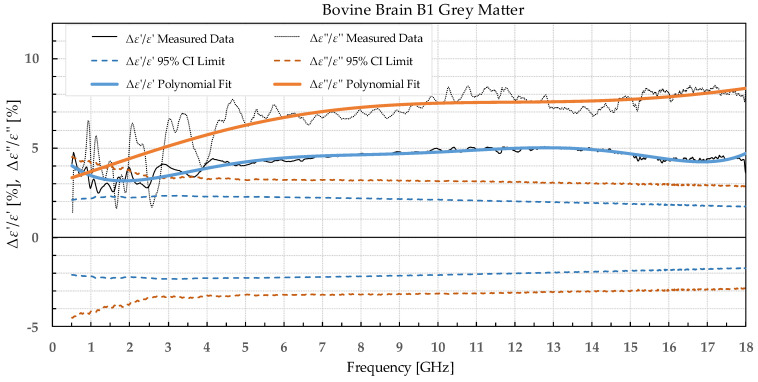
Percent change in *ε*′ and *ε*″ for bovine grey matter before freezing below −18 °C in a commercial freezer and after thawing.

**Figure 3:**
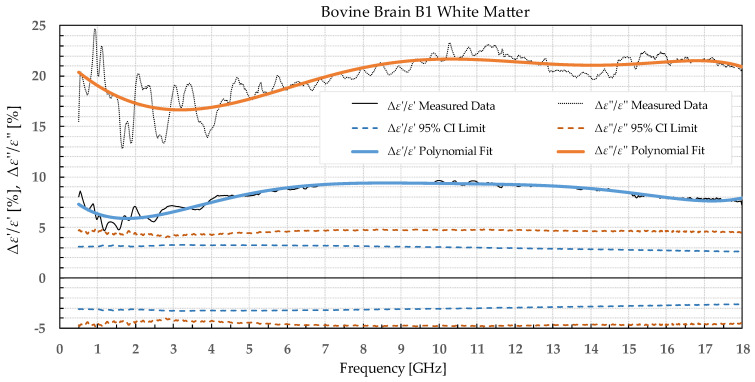
Percent change in *ε*′ and *ε*″ for bovine white matter before freezing below −18 °C in a commercial freezer and after thawing.

**Figure 4:**
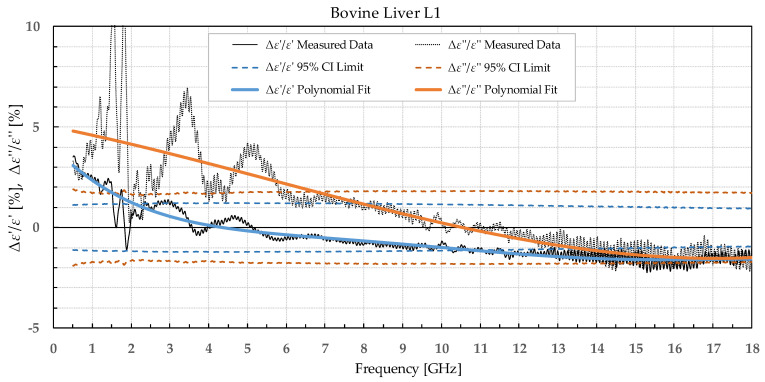
Percent change in *ε*′ and *ε*″ for bovine liver before freezing below −18 °C in a commercial freezer and after thawing.

**Figure 5:**
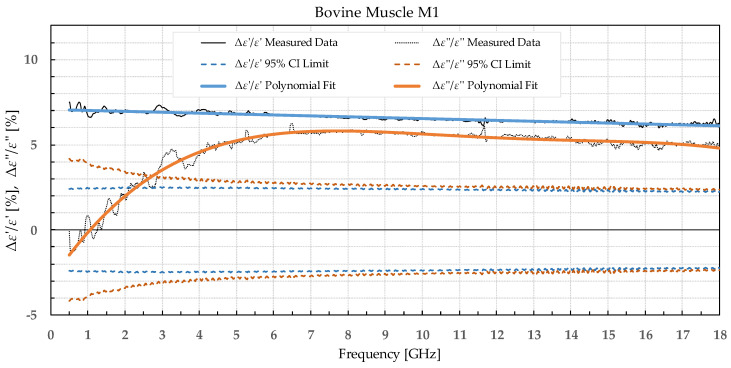
Percent change in *ε*′ and *ε*″ for bovine muscle before freezing below −18 °C in a commercial freezer and after thawing.

**Figure 6:**
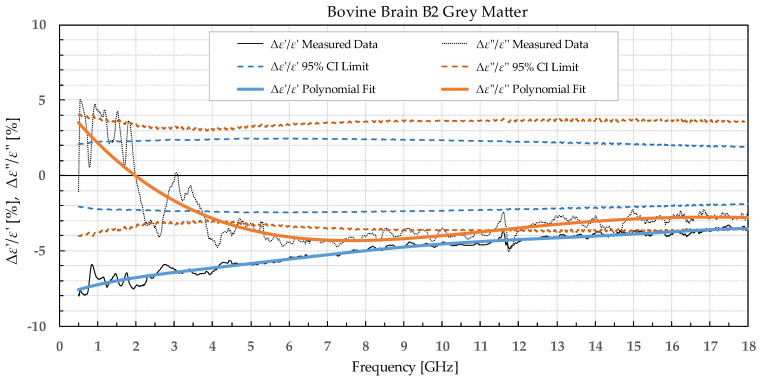
Percent change in *ε*′ and *ε*″ for bovine grey matter before freezing in liquid nitrogen and after thawing.

**Figure 7:**
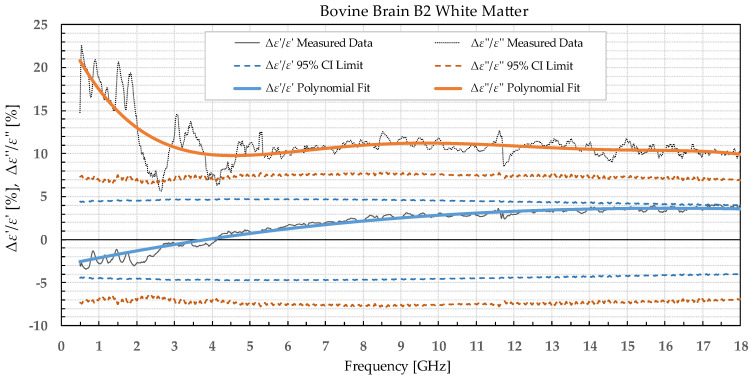
Percent change in *ε*′ and *ε*″ for bovine white matter before freezing in liquid nitrogen and after thawing.

**Figure 8:**
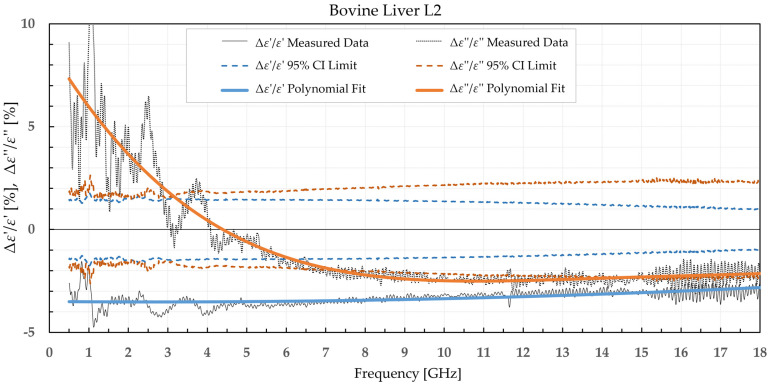
Percent change in *ε*′ and *ε*″ for bovine liver before freezing in liquid nitrogen and after thawing.

**Figure 9:**
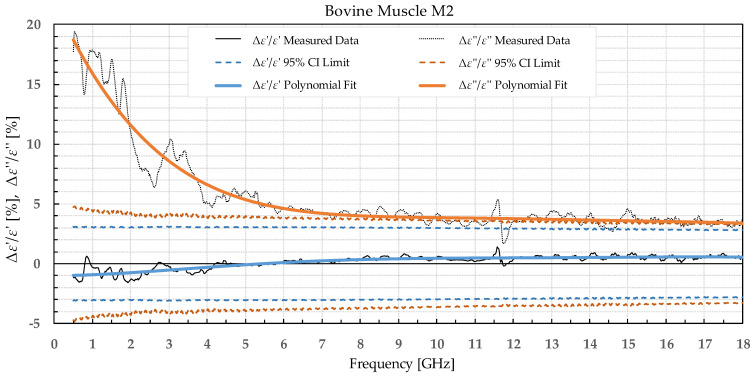
Percent change in *ε*′ and *ε*″ for bovine muscle before freezing in liquid nitrogen and after thawing.

The results were separated into two categories: the percent permittivity change after freezing in a commercial freezer for samples B1, L1, and M1, and percent permittivity change after freezing in liquid nitrogen for samples B2, L2, and M2. The percent change was chosen over the absolute change, as the permittivity greatly varied with frequency; thus, the absolute change would not provide the information on the significance of the change. Additionally, polynomial fitting was performed to smooth out the narrowband fluctuations, which occurred due to the imperfections of the setup and did not reflect the actual change in permittivity due to the freezing process. Raw data are shown by the black solid or dotted line in Figure 2, Figure 3, Figure 4, Figure 5, Figure 6, Figure 7, Figure 8 and Figure 9 for *ε*′ and *ε*″, respectively. The polynomial fits of the raw data in Figure 2, Figure 3, Figure 4, Figure 5, Figure 6, Figure 7, Figure 8 and Figure 9 are shown in a blue solid line for the change in *ε*′ and an orange solid line for the change in *ε*″. Additionally, we added the limits of a statistical test to the charts for easier assessment of statistical significance. Dashed lines of the matching colors represent the limits of the *t*-test with a 95% confidence interval (CI). The actual test is described in Section 3.3.

### 3.1. B1, L1, and M1 Samples

Bovine brain B1 was measured on 17 slices that included a total of 72 measurement points for white matter and 70 points for grey matter before freezing to −18 °C, 73 measurement points for white matter, and 76 points for grey matter after thawing. Bovine liver L1 was measured on 15 different samples at 68 different points before freezing. The measurements on the 15 liver samples already showed that the difference between the liver samples was very small; thus, measuring the additional samples would not significantly increase the statistical strength. Therefore, the rest of the liver samples were frozen without measuring them to minimize the time of sample storage before freezing them. After thawing, 25 different liver samples were measured with 110 measurement points total. The bovine muscle M1 was measured on 21 different samples at 105 measurement points before freezing and 17 samples with 85 measurement points after thawing. The percent change in *ε*′ and *ε*″ of the bovine tissues is presented in Figure 2, Figure 3, Figure 4 and Figure 5.

### 3.2. B2, L2, and M2 Samples

Bovine brain B2 was measured on six slices that included 38 points for white matter and 32 points for grey matter, both before freezing at −196 °C and after thawing. Bovine liver L2 was measured on 10 different samples at 50 different points both before freezing in liquid nitrogen and after thawing back to 25 °C. Bovine muscle M2 was measured on 14 different samples totaling 80 different points both before freezing and after thawing. Figure 6, Figure 7, Figure 8 and Figure 9 present the percent difference in complex permittivity for the bovine tissues frozen in liquid nitrogen. Additionally, we added the limits of a statistical test to the charts for easier assessment of statistical significance. The actual test is described in the next subsection.

### 3.3. Statistical Analysis

We used the unpaired *t*-test to find the difference between the means of the two samples. Because the number of samples was not considered small, i.e., was over 30, the samples did not need to follow the normal distribution (although we expected that they actually would do). An additional requirement was that the two populations do not differ in the standard deviation, which was also satisfied, as shown in Table 1 which reports the standard deviation averaged over the entire measured frequency range. T-values were calculated for each frequency point of each tissue type and for each freezing method. The calculated values were compared to the values from the table of critical t-values chosen for a two-tailed test with a significance level of *α* = 0.05, i.e., the confidence interval of 95% [11]. The limits of the decision criterion for statistical significance in the change of permittivity with the used 95% CI *t*-test are represented with dashed lines in Figure 2, Figure 3, Figure 4, Figure 5, Figure 6, Figure 7, Figure 8 and Figure 9.

## 4. Discussion

The agreement of our initial permittivity measurements prior to freezing the tissues with the previously published data [12,13] is satisfactory. The permittivity of the bovine liver and muscle has been measured in [12] from 500 MHz to 40 GHz at 37 °C. Even though our measurements were performed at 25 °C, due to the lack of the reported data for our specific tissue type, animal species, measurement frequency, and tissue temperature, we used their data for control and comparison. Our results for the bovine liver and muscle are comparable to the results in [12], taking into account that their measurements were performed at 37 °C. Bovine brain white matter and grey matter have been measured in [13] at 35 °C. The measured bovine brains were separated into two groups: adult animals, i.e., 16–24 months, and young animals, i.e., 4–6 months. Again, due to the lack of a more similar experimental setup and samples, we used their results for control and comparison. The results for both the grey and white matter are comparable to [13] for young calves when corrected for the difference in temperature.

As our preliminary results [5] indicated that freezing in a commercial freezer did, in fact, change the dielectric properties of the tissues, we also explored other freezing methods with faster freezing. Both methods of cooling the tissue to freezing temperatures and warming the tissue during thawing can cause the formation of damaging ice crystals. The ice formation during either cooling or warming differs between the methods, depending on how much time a method allows for the ice crystals to generate and grow. Therefore, it is possible to have a method with a sufficiently high cooling and warming rate where the ice formation will be suppressed [14]. That is why we also explored an additional freezing method by submerging the tissue samples in liquid nitrogen. Freezing by immersing in liquid nitrogen causes the process of freezing to happen much quicker than in a commercial freezer. Thus, by using two different freezing methods, in a commercial freezer and in liquid nitrogen, the effects of both slower and faster cooling rates on the sample permittivity were explored. During slow freezing, the extracellular water turned to ice, which is an almost completely pure substance, leaving the dissolved solutes in a reduced volume of solvent. The process inevitably changes the osmotic pressure and causes the intracellular water to flow outside the cell to restore the osmotic equilibrium, essentially dehydrating the cell and changing the permittivity [14]. Freezing with higher cooling rates should, in theory, prevent the described dehydration mechanism. However, cooling too quickly could cause intracellular ice formation that could rupture the membrane and lead to cell death. Between these two ends of the spectrum, there is an optimum cooling rate that minimizes both sources of freeze injury. Usually, cryopreservation is paired with cryoprotective agents to further reduce the damaging effects of frozen tissues during rapid freezing [15]. Unfortunately, the use of cryoprotectants on tissue samples would alter the dielectric properties, whereas the objective of this research was to find a way to preserve the permittivity, which is why our freezing protocol did not include the use of cryoprotectants.

The statistical analysis showed that the difference in permittivity before freezing and after thawing was not negligible. Overall, the most suitable tissue for freezing is the liver tissue which exhibited the smallest percent permittivity change for both freezing protocols. The most significant difference was observed for the white matter, where the difference in *ε*″ exceeded 20% at some frequency points.

The permittivity of both white and grey matter B1 displays a significant difference before and after freezing in the whole measured frequency range. The exception to this is *ε*″ below ca. 1 GHz, but this is practically insignificant. Freezing the brain B2 in liquid nitrogen and thawing caused a significant difference over the whole measured frequency range in *ε*″ of the white matter and *ε*′ of the grey matter. *ε*′ of the white matter did not display significant change over the whole frequency range, while the *ε*″ of the grey matter sporadically lingered around the set confidence interval limit at frequencies over 4.5 GHz.

When analyzing liver permittivity results, *ε*′ of L1 did not display a significant change in the 2–11 GHz range, while *ε*″ displayed a significant difference for frequencies under 7 GHz. The liver L2 had a significant decrease in *ε*′ over the whole measured range, while *ε*″ sporadically exceeded the chosen confidence interval and associated t-value under 3.5 GHz, between 7.5 and 13.5 GHz, and over 17.5 GHz.

Bovine muscle M1 displayed a significant increase in *ε*′ over the whole measured frequency range, while *ε*″ showed a significant change over ca. 3 GHz. For the muscle frozen and stored in liquid nitrogen, M2, there were no significant differences in *ε*′, while *ε*″ exhibited an increase over the whole frequency range but was especially prominent at the lowest measured frequencies.

Freezing the tissues in liquid nitrogen results in a decrease in *ε*′ when compared to the same tissues frozen in a commercial freezer. The trends are not consistent between different tissues frozen with the same method. It was already observed in [16] that different tissues undergo different physical processes during a freeze–thaw cycle, which could also explain the difference in permittivity trends. Their preliminary investigations indicated that some tissues, such as the brain or spleen, soften after thawing because they have weak intercellular junctions or high extracellular water content and, therefore, lose their structure after thawing. Other tissues, such as skin or skeletal muscle, harden because their cells lose water and are unable to restore that water after thawing.

Comparing our measured permittivity difference for muscle and liver tissue before and after freezing with a similar study [7], there are several consistent observations. In their work, the permittivity of muscle tissue increased after thawing for both *ε*′ and *ε*″ by 2.1% and 6.4% at 4.5 GHz, respectively. In our study, the increase in permittivity was 7% for *ε*′ and 5% for *ε*″ for muscle frozen in a commercial freezer measured at 4.5 GHz. The trend of increase in permittivity was consistent between the studies, and both studies reported significant differences in both *ε*′ and *ε*″. The difference in percentage could be explained by the different compositions of the chicken versus the bovine muscle. Their results for bovine liver showed an increase in permittivity by 2.6% for *ε*′ and 8.9% for *ε*″ at 4.5 GHz. Our results at 4.5 GHz showed that the liver L1 had almost no change in *ε*′ and 3% in *ε*″. Even though at first it appears that the results do not fully agree, it is worth noting that the statistical analysis of liver permittivity results in [7] found that there is a significant difference for *ε*″ but not for *ε*′, and the same was observed in our study (Figure 4).

Unfortunately, unlike the muscle and liver tissue, which were measured before and after freezing in a similar study [7], there is no similar study on freezing the brain tissues to compare our results to.

## 5. Conclusions

To our knowledge, this is the first measurement study of permittivity change in brain tissue due to a freeze–thaw cycle. Our results suggest that both of the tested freezing methods produce a statistically significant change in permittivity, at least in some part of the measured frequency range. Nevertheless, depending on the application and the preferred frequency range, the permittivity change might be acceptable. If the acceptable percent change is set to, e.g., 10%, the measured permittivity change is satisfactory for liver, muscle (but only when frozen in a commercial freezer), and grey matter, while the permittivity change in white matter and muscle frozen in liquid nitrogen is not acceptable, but only due to the change in *ε*″ which exceeds the set limit.

The necessity for finding a tissue preservation method that would not significantly influence the complex permittivity still remains.

## Figures and Tables

**Table 1:** Average standard deviations of *ε*′ and *ε*″ for bovine tissues.

	Tissue Type	Before Freezing		After Thawing	
		*ε*′	*ε*″	*ε*′	*ε*″
Freezer	White Matter	3.11	1.53	2.54	1.39
	Grey Matter	3.18	2.13	2.44	1.59
	Liver	1.29	0.88	1.36	0.97
	Muscle	3.80	1.99	3.21	2.04
Liquid Nitrogen	White Matter	2.15	0.99	3.54	1.95
	Grey Matter	1.74	1.00	2.30	1.57
	Liver	0.93	0.47	1.52	1.05
	Muscle	4.42	2.57	3.63	2.20

## Data Availability

Not applicable.
